# Network topology of the marmoset connectome

**DOI:** 10.1162/netn_a_00159

**Published:** 2020-12-01

**Authors:** Zhen-Qi Liu, Ying-Qiu Zheng, Bratislav Misic

**Affiliations:** McConnell Brain Imaging Centre, Montréal Neurological Institute, McGill University, Montréal, Quebec, Canada; Biomedical Engineering, University of Oxford, Oxford, United Kingdom; McConnell Brain Imaging Centre, Montréal Neurological Institute, McGill University, Montréal, Quebec, Canada

**Keywords:** Marmoset, Connectome, Brain connectivity, Motif, Persistent homology

## Abstract

The brain is a complex network of interconnected and interacting neuronal populations. Global efforts to understand the emergence of behavior and the effect of perturbations depend on accurate reconstruction of white matter pathways, both in humans and in model organisms. An emerging animal model for next-generation applied neuroscience is the common marmoset (*Callithrix jacchus*). A recent open respository of retrograde and anterograde tract tracing presents an opportunity to systematically study the network architecture of the marmoset brain (Marmoset Brain Architecture Project; http://www.marmosetbrain.org). Here we comprehensively chart the topological organization of the mesoscale marmoset cortico-cortical connectome. The network possesses multiple nonrandom attributes that promote a balance between segregation and integration, including near-minimal path length, multiscale community structure, a connective core, a unique motif composition, and multiple cavities. Altogether, these structural attributes suggest a link between network architecture and function. Our findings are consistent with previous reports across a range of species, scales, and reconstruction technologies, suggesting a small set of organizational principles universal across phylogeny. Collectively, these results provide a foundation for future anatomical, functional, and behavioral studies in this model organism.

## Author Summary

Emerging research points to network connectivity as a fundamental feature of brains, influencing interregional signaling, cognition, and behavior. Global efforts are under way to map, image, and trace cellular connection patterns in humans and in a variety of model organisms. The common marmoset is increasingly becoming the most used nonhuman primate model for neuroimaging, genomics, and behavioral research. Here we provide a comprehensive characterization of the marmoset brain connectome using a recently published cortico-cortical tract tracing atlas. We find evidence of nonrandom organization across multiple scales, including near-minimal path length, multiscale community structure, densely interconnected hubs, a unique motif fingerprint, and the existence of topological cavities. Collectively, these features suggest that the network is configured to support the coexistence of segregation and integration of information.

## INTRODUCTION

The brain is a networked system of distributed neuronal populations. The complex arrangement of anatomical projections supports interregional signaling and functional interactions, manifesting as patterned neural activity. Collective signaling in the network is thought to support perception, cognition, and action. Recent technological advances permit extensive tracing and imaging of neural circuits in humans and nonhuman model organisms.

The composition and architecture of brain networks reveals potential routes and mechanisms by which signals are transmitted and integrated (Avena-Koenigsberger, Misic, & Sporns, 2018; Suárez, Markello, Betzel, & Misic, 2020). Numerous studies of structural networks have revealed characteristic network features such as high local clustering and short path length (Kaiser & Hilgetag, 2006), as well as the existence of functionally specialized modules interlinked by densely connected and highly central “hubs” (Betzel et al., 2013; Hilgetag & Kaiser, 2004). Hubs have a pronounced tendency to be interconnected with each other (van den Heuvel, Kahn, Goñi, & Sporns, 2012), forming an integrated core (Zamora-López, Zhou, & Kurths, 2010). These organizational attributes have been reported in nervous systems of a range of species, suggesting common organizational principles across phylogeny (Goulas, Majka, Rosa, & Hilgetag, 2019; van den Heuvel, Bullmore, & Sporns, 2016). Mapping the architecture of neural circuits to behavioral and disease phenotypes remains a key challenge for the field (Mišić & Sporns, 2016).

An emerging animal model for next-generation human clinical and behavioral neuroscience is the common marmoset (*Callithrix jacchus*; Okano et al., 2016). A New World monkey, the marmoset shares many key traits with humans, including complex individual and social behavior (Miller et al., 2016; Yokoyama & Onoe, 2015). Anatomically, marmoset cortex and subcortex are also characterized by complex connection patterns, extensive white matter, and a well-developed, granular frontal cortex (Reser et al., 2017). Short gestation times accelerate cross-generation studies (Burkart & Finkenwirth, 2015). With the advent of transgenic modification techniques and high-field imaging (Kishi, Sato, Sasaki, & Okano, 2014), the marmoset is poised to become a powerful model for neurodevelopment and neurodegeneration, for monitoring pathophysiological progression, and for testing therapeutic interventions in vivo.

The goal of the present report is to compile a complete quantitative topological characterization of the marmoset white matter connectome. We use state of the art retrograde and anterograde tract tracing atlas from the Marmoset Brain Architecture Project (Majka et al., 2016). We then systematically investigate a range of topological attributes and organizational principles thought to be important for neural signaling and communication, including hub distribution, community structure, motif composition, and higher order organization.

## RESULTS

Tracer data were downloaded from the Marmoset Brain Architecture Project (http://www.marmosetbrain.org/; Majka et al., 2016). The directed network comprises 55 cortical nodes and 1,861 directed edges. Injection sites span the cortex (Paxinos, Watson, Petrides, Rosa, & Tokuno, 2011), including frontal cortex, motor and premotor cortex, somatosensory cortex, posterior parietal cortex, medial and retrosplonial cortical areas, auditory cortex, visual cortex, part of lateral and inferior temporal cortical regions, part of orbital frontal cortex, and part of posterior cingulate cortex. For further details about network reconstruction, please see the Materials and Methods section.

### Global Topological and Geometric Attributes

**Figure 1.**
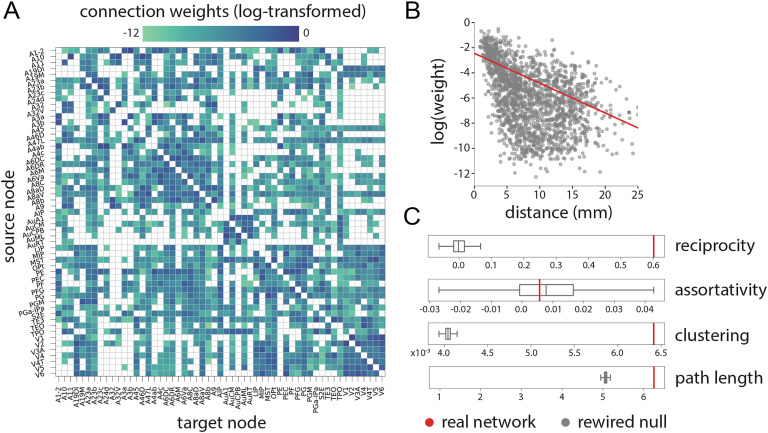
Global topological and geometric attributes. (A) The FLNe-labeled marmoset cortico-cortical connectome. Nodes are *n* = 55 cortical regions with complete input and output projections, giving a sparse weighted directed network (rows = source areas, columns = target areas) with *k* = 1,861 edges. The weights are log-transformed. (B) Connection weight (y-axis) versus spatial separation (x-axis). Each point represents a projection. (C) Global topological attributes of the network, including reciprocity (tendency for nodes to be mutually linked), assortativity (tendency for nodes to link with similar nodes), clustering (proportion of completed triangles around a node), and path length (mean shortest set of edges connecting all pairs of nodes in the network). Network measures are shown in red; distributions from 1,000 in-degree and out-degree preserving rewired null networks are shown in gray.

With 55 nodes and 1,861 directed edges, the network is relatively dense (connection density: 0.626, directed, and 0.601, undirected), consistent with recent reports on the macaque (Goulas et al., 2014; Markov et al., 2012, 2013, 2014). The weight of connections between areas decreases monotonically with spatial separation, and the decrease is roughly exponential (Figure 1B) (Horvát et al., 2016; Mišić, 2014; Roberts et al., 2016). The strength and binary degree distributions are right-skewed, suggesting the existence of disproportionately well-connected nodes or hubs and thus a potentially nonrandom organization (Figure S1).

The network possesses similar connection weights bidirectionally (reciprocity r = 0.602), several magnitudes greater than in rewired null networks (*r*_*rand*_ = 0.001; Figure 1C), suggesting a tendency for bidirectional communication. The strength assortativity of the network is positive (0.006) but close to 0 and indistinguishable from the null distribution, suggesting no evidence of homophilic attachment among nodes with similar strengths. The network is highly clustered (clustering coefficient 0.006; normalized clustering coefficient *γ* = 1.586) with short characteristic path length (characteristic path length 6.25, normalized characteristic path length *λ* = 1.23; Figure 1C). Thus it exhibits the small-world property (small-world index *σ* = 1.29), consistent with previous findings across multiple model organisms (Hilgetag, Burns, O’Neill, Scannell, & Young, 2000; Watts & Strogatz, 1998; but see also Hilgetag & Goulas, 2016).

### Community Structure

We next seek to characterize the mesoscale structure of the network: the architectural properties that describe the tendency for neural elements to be organized into circuits and clusters. By decomposing the network into functionally meaningful components, it becomes possible to infer node- and edge-level involvement in the network at multiple topological scales.

**Figure 2.**
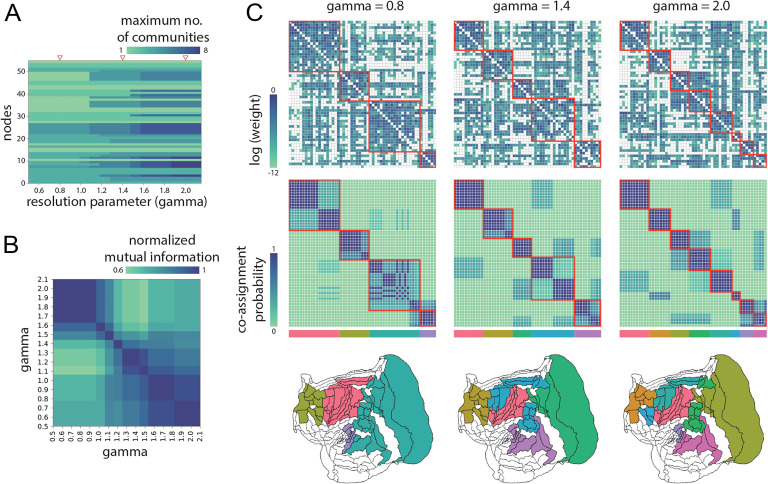
Community structure. A greedy Louvain-like algorithm was used to identify network communities. (A) The algorithm was run 1,000 times at multiple resolutions (*γ* parameter), yielding potential community assignments at multiple scales. (B) Normalized mutual information among partitions suggests three prominently defined scales, centered on *γ* = 0.8, *γ* = 1.4, and *γ* = 2.0 (indicated by red triangles in panel A). (C) Consensus community partitions at each scale, revealing 4, 5, and 8 communities. Top row: adjacency matrix reordered by community assignment and strength. Middle row: co-assignment probability across 1,000 runs of the Louvain algorithm. Bottom row: flatmap projections of community assignments.

We use multiscale community detection to describe the modular organization of the network. We apply a Louvain-like greedy modularity maximization method and perform a parameter search across a range of resolutions (*γ* = 0.5…2.0); the search allows us to tune the sensitivity of the algorithm from a small set of large communities (low resolution) to a large set of small communities (high resolution) (Figure 2A). We use normalized mutual information to evaluate patterns of similarity between partitions over resolutions *γ* (Figure 2B). The procedure identifies three regimes at which community structure is well defined, revealing a nested hierarchical architecture (Figure 2).

We focus on three points in parameter space (*γ* = 0.8, *γ* = 1.4, and *γ* = 2.0; shown in red triangles in Figure 2A). The partitions yielded 4, 5, and 8 spatially contiguous communities, respectively. For the three resolutions, Figure 2C shows the consensus partition (top), the frequency of co-assignment across multiple algorithm runs (middle), and the spatial layout (bottom). Across resolutions, the frontal cortex is consistently identified as a community, including dorsolateral and ventrolateral aspects. At the highest resolution (*γ* = 2.0), the ventrolateral aspect becomes a separate community. The auditory cortex is also well delineated as a standalone community. At the lowest resolution, the remaining areas are distributed among two communities; one mainly contains motor and premotor cortical regions and somatosensory cortex, while the other mainly contains visual areas and parts of lateral and inferior temporal cortex. At higher resolutions these communities are further broken up into more specialized units, such as ventrolateral frontal cortex or inferior temporal cortex (Hung et al., 2015). Altogether, the analysis highlights the existence of a multiscale community structure that maps onto increasingly specialized functional domains in the marmoset brain.

### Hub Organization

In the previous section we demonstrated that the marmoset connectome displays functionally meaningful community organization at multiple scales. This feature is thought to promote segregation of streams of information, helping to concentrate communication within specialized neural circuits (Hilgetag et al., 2000; Hilgetag & Kaiser, 2004). At the same time, coherent perception, cognition, and action necessitate a complementary integrative mechanism. Hubs—central nodes with diverse connection profiles—are thought to be a fundamental feature that allows information to be sampled and ultimately integrated from multiple specialized communities (Zamora-López et al., 2010).

**Figure 3.**
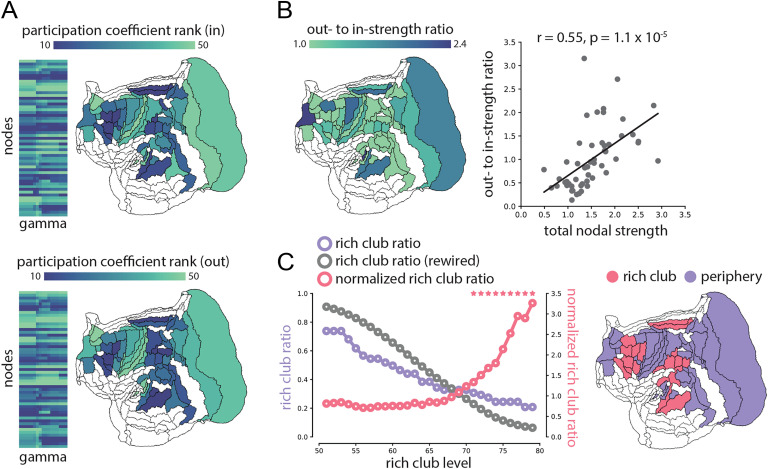
Hub organization. (A) The mean rank-transformed participation coefficients across the range of resolution parameters. In-degree participation coefficients quantify the diversity of a node’s afferent projections; out-degree participation coefficients quantify the diversity of a node’s efferent projections. (B) Out-strength to in-strength ratio, showing nodes with greater than expected efferent connectivity. The ratio is correlated with total node strength, meaning that densely connected nodes are more likely to have greater than expected out-strength. (C) Rich club detection was performed by computing the density of subgraphs composed of ≥ *k* nodes (rich club ratio; purple), and comparing it with densities of 1,000 degree-preserving randomly rewired networks (rewired rich club ratio; gray). The ratio between the two (normalized rich club ratio; pink) indicates levels where high-degree nodes in the empirical network are significantly more connected with each other than expected on the basis of their degree alone. *P* values are estimated as the proportion of rewired rich club ratios that are greater than the empirical rich club ratio (asterisks). Rich and nonrich (peripheral) nodes projected on a flatmap.

We begin by studying the diversity of a node’s connections (participation coefficient; Guimera & Amaral, 2005). Values close to 0 indicate that a node is exclusively connected to other members of the same module, while values close to 1 indicate that a node’s connections are evenly distributed among multiple modules. For a particular resolution, we calculate the participation coefficient for every node and rank nodes according to their participation coefficients. We then average the ranks of participation coefficients over all partitions (Figure 3A). Node participation ranks are consistent across resolutions (Figure 3A; heatmaps), suggesting that nodes tend to act as hubs across a range of scales. For both in- and out-projections, nodes with the greatest participation were located in medial frontal cortex, auditory cortex, and visual cortex. Node participation is also correlated with node degree (in-participation: *r* = 0.69, *p* = 4.7 × 10^−9^; out-participation: *r* = 0.76, *p* = 2.75 × 10^−11^), suggesting that densely connected nodes tend to have diverse connection profiles (Rubinov, Ypma, Watson, & Bullmore, 2015).

The resolved directionality in the tracer-labeled marmoset connectome allows us to consider whether some areas are more likely to act as sources or sinks for information flow (Markov et al., 2013; Mišić, Goñi, Betzel, Sporns, & McIntosh, 2014; van den Heuvel, Scholtens, & de Reus, 2016). To address this question, we compute the ratio of out-strength to in-strength for each node and relate this quantity to total strength (Figure 3B). We note two results. First, the range of out- to in-strength ratios is considerable, suggesting that some areas are better positioned to absorb signal traffic, while others are better positioned to disseminate signal traffic. Second, total strength and out- to in-strength ratios are correlated (*r* = 0.55, *p* = 1.1 × 10^−5^), meaning that nodes with greater strengths are also more likely to act as sources rather than sinks. There are also several notable outliers with disproportionately large out-strength than expected on the basis of their overall strength, including frontal polar A10 and inferior temporal area TE3 (Figure 3C).

We finally investigate the relationship among the well-connected nodes. An oft-observed phenomenon in brain networks is that high-degree hubs tend to be disproportionately connected with each other (Zamora-López et al., 2010), forming a putative communication backbone (van den Heuvel et al., 2012). This tendency can be statistically detected and quantified using the rich club framework, whereby the connection density of subgraphs containing only nodes with degrees ≥ *k* is compared against an ensemble of rewired null networks (Colizza, Flammini, Serrano, & Vespignani, 2006; Opsahl, Colizza, Panzarasa, & Ramasco, 2008). Figure 3C shows the density of subgraphs composed of nodes with increasing degree (purple), the density of subgraphs for rewired networks (gray), and the ratio between the two (pink). At *k* ≥ 70 the ratio between the real network and rewired nulls is consistently and significantly greater than 1, suggesting evidence of rich club organization. Anatomically, rich club nodes are evenly distributed throughout the marmoset brain, including portions of frontal cortex, motor cortex, auditory cortex, and inferior temporal cortex (A23a, A23b, A45, A47L, A6DC, A6DR, A6Va, A8aD, A8aV, A8b, AIP, AuCM, MST, OPt, PFG, PG, S2E, TE3, TPO). Notably, rich club nodes are evenly distributed among the segregated communities, providing an infrastructural feature to sample and integrate information from specialized domains.

### Higher Order Interactions

Finally, we study the propensity of the network to support higher order interactions. So far we have focused on network attributes derived from dyadic (two node) relationships, but an emerging literature emphasizes the role of higher order interactions that simultaneously take place among more than two nodes, including motifs and cavities (Benson, Gleich, & Leskovec, 2016; Sizemore, Phillips-Cremins, Ghrist, & Bassett, 2019).

**Figure 4.**
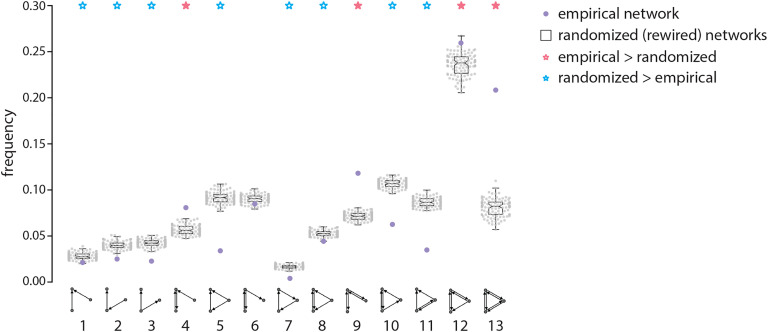
Motif composition. The prevalence of 13 distinct three-node motif arrangements is quantified in the empirical network (purple) and in a population of 100 degree-preserving randomly rewired networks (black boxplots). Two-tailed tests indicate that some motifs are significantly more frequent in the empirical network (motifs 4, 9, 12, 13; pink asterisk) and some are significantly less frequent (motifs 1, 2, 3, 5, 7, 8, 10, 11; blue asterisk), suggesting that the topology of the marmoset connectome possesses a unique motif fingerprint, promoting specific nonrandom circuit configurations. Boxplot whiskers represent the most extreme, non-outlier data points (≤ 1.5 × inter-quartile range).

We first consider the motif composition of the network (Milo et al., 2002; Shen et al., 2012; Sporns & Kötter, 2004). We focus only on three-node motifs as they can take 13 distinct configurations (four-node motifs can take 199 combinations, making the analysis of larger motifs computationally prohibitive). Specifically, we study the frequency with which motifs occur and compare this with an ensemble of rewired null networks (Figure 4; real network frequencies = purple; null network frequencies = black). Comparison to the null model reveals that the network is enriched for several specific motifs (denoted by red stars; *p* ≤ 0.05), including motifs 4, 9, and 13. The network is also deficient in several motifs (denoted by blue stars; *p* ≤ 0.05), including motifs 1–3, 5, 10, and 11. This motif spectrum is largely consistent with previous reports on the macaque (Shen et al., 2012; Sporns & Kötter, 2004). The relative abundance of motif 9 is noteworthy, as it suggests the existence of “chains” of reciprocally connected regions, without connections between the ends of the chains. This suggests a functional tendency to balance segregation and integration: Areas are integrated with their neighbors, but some pairs of areas have no direct connection and are therefore segregated from each other (Sporns & Kötter, 2004).

**Figure 5.**
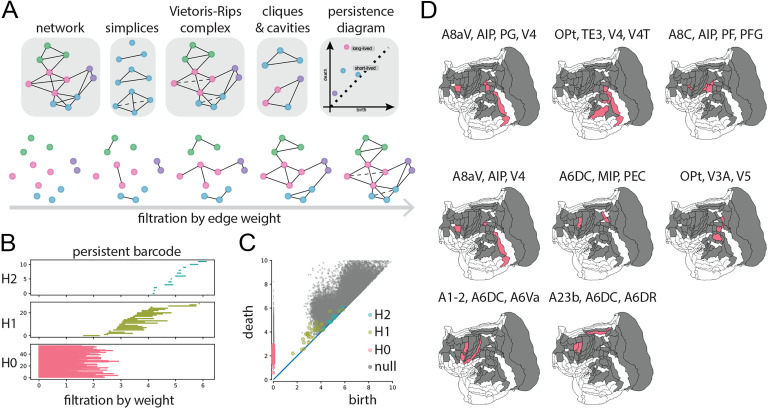
Higher-order organization. (A) Edge filtration was used to identify the appearance of persistent homology. We focused on topological cavities: circuit configurations that promote serial or sequential passing of information among constituent nodes. (B) Edge filtration applied to the marmoset network, focusing on 2D and 3D cavities (H1 and H2, respectively). (C) Edge filtration identified multiple long-lived cavities in the empirical network (red, green, blue), but generally fewer than in randomized networks (gray). (D) Representative three-and four-node cavities projected onto a flatmap.

Finally, we investigate the spectrum of higher order structures in the network (Giusti, Ghrist, & Bassett, 2016; Sizemore et al., 2018, 2019). In particular, we focus on connections that are unexpectedly absent from the network, suggesting attenuation of communication. The goal of the analysis is to identify topological cavities: clusters of nodes that form voids, precluding parallel information exchange and promoting serial information exchange instead. We first apply edge weight filtration: beginning with the empty graph and adding one edge at a time, in order of decreasing weight (Figure 5A). Through the filtration process, we track the appearance and disappearance of topological cavities. The process yields a persistence “barcode” (Figure 5B) and diagram (Figure 5C), representing cycles as they appear (birth) or disappear (death) through the filtration process. Each bar (row) in Figure 5B corresponds to one point in Figure 5C, giving an intuitive overview of the cycles’ lifetime. Cavities here are represented by their minimal representative cycles just before death time, and are referred to using this definition. *H* subscripts represent cavities of increasing dimensions; H0 represents connected components, H1 represents 2D holes, and H2 represents 3D holes (see Figure 5A). Cavities that persist for a long time (defined as death time minus birth time) represent encapsulated combinations of neuronal populations where information is not exchanged in parallel, but rather in a serial fashion over extended paths. The existence of persistent cavities is then compared with a rewired null model (Figure 5C, gray).

Compared with a population of randomly rewired networks, the marmoset network displays multiple statistically unexpected cavities (Figure 5C). Figure 5D shows the top three cavities with four nodes and top five cavities with three nodes, ranked by persistence. Of the four-node cavities, one spans dorsolateral prefrontal cortex, posterior parietal cortex, and visual cortex (A8aV, AIP, PG, V4), one is located mostly in the posterior half of the brain of posterior parietal cortex, inferior temporal cortical region, and visual cortex (OPt, TE3, V4, V4T), and one mainly resides in posterior parietal cortex, with one node in the premotor cortex (A8C, AIP, PF, PFG). In general, the cavities are distributed across the processing hierarchy, typically including both primary sensory-motor areas and higher order polysensory areas, suggesting the existence of compact circuits that support serial trans-hierarchical processing.

## DISCUSSION

The present report comprehensively characterizes the mesoscale connectome of the marmoset brain. Using high-quality tracer data, we demonstrate that the marmoset brain possesses multiple nonrandom attributes, including multiscale community structure, a connective core, a unique motif fingerprint, and multiple cavities. These organizational principles are concordant with numerous reports in other model organisms, highlighting conserved architectural features across phylogeny.

The configuration of the marmoset connectome suggests a balance between features that promote segregation and those that promote integration of information (Sporns, 2013). The network is highly clustered (Figure 1), with dense connectivity among areas that participate in similar functions, manifesting as a nested hierarchy of specialized communities or modules (Figure 2). Connection patterns bypass some combinations of areas, yielding topological cavities that effectively attenuate signaling in specific circuits (Figure 5). Collectively, these features ensure that signals are exchanged within specific components of the network, limiting exposure to and interference from signals originating elsewhere in the network.

At the same time, the network also possesses several disproportionately connected areas that provide short communication paths between pairs of areas, resulting in near-minimal path length (Figure 1). High-degree “hubs” are distributed among communities, possess diverse connectional fingerprints, and are densely interlinked with each other (Figure 3), forming a connective core that allows sampling and integration of information from specialized domains (Zamora-López et al., 2010). Altogether, these findings reveal a computational infrastructure that can simultaneously support a range of localized and distributed operations.

Collectively, these organizational attributes highlight the link between anatomical connectivity and functional domains (Suárez et al., 2020). A salient example is the fact that specialized communities typically encompass unimodal areas, whereas high-participation rich club areas are located in polysensory association cortex. We envision that these findings will help to inform future work on structure-function relationships, including studies of hierarchical organization (Buckner & Margulies, 2019; Theodoni et al., 2020), comparisons of structural and functional connectivity (Hori et al., 2020), and ultimately, large-scale computational models (Shen et al., 2019). Indeed, the marmoset connectome possesses a prominent hierarchical architecture defined by the laminar dependence of connections, which is reflected by the cytological attributes of individual areas (Theodoni et al., 2020).

More generally, these findings contribute to a growing literature on comparative network anatomy (van den Heuvel, Bullmore, et al., 2016). The topological properties of the marmoset cortico-cortical connectome are consistent with reports from a wide range of species, from the invertebrate nematode worm (Towlson, Vértes, Ahnert, Schafer, & Bullmore, 2013; Varshney, Chen, Paniagua, Hall, & Chklovskii, 2011) and the fruit fly (Shih et al., 2015), to multiple avian (Layden, Schertz, London, & Berman, 2019; Shanahan, Bingman, Shimizu, Wild, & Güntürkün, 2013), rodent (Bota, Sporns, & Swanson, 2015; Rubinov et al., 2015; van den Heuvel, Scholtens et al., 2016), feline (de Reus & van den Heuvel, 2013), and nonhuman primate species (Harriger, van den Heuvel, & Sporns, 2012; Markov et al., 2013; Modha & Singh, 2010). Despite differences in acquisition technology, spatial resolution, preprocessing, and sample size, the findings point to a small but highly conserved set of organizational principles.

Cross-species comparisons suggest a common set of network attributes, but which of these attributes are statistically unexpected and what forces shape their emergence (Betzel et al., 2016; Goulas et al., 2019; Rubinov, 2016; Vértes, Alexander-Bloch, & Bullmore, 2014)? The most common inferential procedure used in the field, including the present report, involves comparisons of network attributes with null distributions generated by random edge rewiring (Maslov & Sneppen, 2002). This approach provides a single reference point, precluding comparisons with a wider range of possible network configurations (Gollo et al., 2018; Zamora-López & Brasselet, 2019), and thus limiting inferences about generative mechanisms. We envision that future studies will take a more comprehensive approach, situating biological connectomes in a broader context or morphospace spanned by multiple traits (Avena-Koenigsberger, Goñi, Solé, & Sporns, 2015), and equally importantly, populated by multiple comparable graphs (Zamora-López & Brasselet, 2019). Consistent with the present results, a recent report in the marmoset connectome found that connection placement is strongly driven by spatial embedding, as well as by cytoarchitectural similarity (Theodoni et al., 2020), but more research is needed to understand the generative principles governing the formation of the marmoset connectome.

## METHODS

All graph measures except persistent homology were computed using *Brainconn* (https://github.com/FIU-Neuro/brainconn). Persistent homology was investigated using the Python binding of Dionysus 2 (http://mrzv.org/software/dionysus2/index.html). Code used to perform the reported analyses is available on GitHub (Liu, 2020; https://github.com/netneurolab/marmoset_connectome) under the BSD 3-Clause License.

### Data Acquisition

Data were downloaded from the Marmoset Brain Architecture Project. The digital repository provides a cellular resolution cortico-cortical connectome reconstructed using neuroanatomical tracers (Majka et al., 2016, 2020). The 143 injections of retrograde tracers included in the original data source were performed on 52 young adult marmosets (1.4–4.6 years, median 2.5 years; 31 male, 21 female). Six types of monosynaptic retrograde fluorescent tracers were applied at multiple injection sites. For details about surgical and histological processing, see Majka et al. (2020). Connection weights are quantified by the extrinsic fraction of labeled neurons (FLNe), representing the proportion of labeled neurons found in the target area to the total number of labeled neurons excluding the neurons in the injected area (Markov et al., 2014). The resulting data are preserved, analyzed with a standardized pipeline, and mapped to the Paxinos stereotaxic atlas using expert-assisted registration (Paxinos et al., 2011).

### Network Preparation

Brain areas were defined according to the Paxinos marmoset atlas (Paxinos et al., 2011). Three-dimensional coordinates for the injection sites are obtained and averaged to get the injection center in each area. A directed FLNe-weighted structural connectivity matrix was constructed by including only nodes with pairwise-complete connection values (*N* = 55), resulting in a 55 × 55 weighted directed adjacency matrix with *k* = 1,861 edges. Zero-valued elements in the adjacency matrix indicate that areas are not connected, as opposed to not tested. Interareal distances were estimated as the Euclidean between injection centroids of each area.

### Local and Global Properties

Local and global measures were implemented in *Brainconn* (https://github.com/FIU-Neuro/brainconn), a derivation of *bctpy* (https://github.com/aestrivex/bctpy), which implements Python version of Brain Connectivity Toolbox (Rubinov & Sporns, 2010). The weighted analogues of node in- and out-degree (in-strength and out-strength) were defined as the sum of all afferent and efferent weighted connections (*w*_*ij*_) at a node, respectively:

$$s_{i}^{in}=\Sigma_{j}w_{ji}\;\text{and}\;s_{i}^{out}=\Sigma_{j}w_{ji}.$$ (1)

At the global level, reciprocity was used to measure the similarity of directed connections. For weighted networks, we use the definition from Garlaschelli and Loffredo (2004), which is calculated as the correlation coefficient between the adjacency matrix and its transpose:

$$\rho =\frac{\Sigma_{i\neq j}(w_{ij}-\overset{-}{w})(w_{ji}-\overset{-}{w})}{\Sigma_{i\neq j}(w_{ij}-\overset{-}{w})\Sigma_{i\neq j}(w_{ji}-\overset{-}{w})},$$ (2)

where $\overset{-}{w}$ denotes the sample mean.

To measure the preference for nodes to connect to others with similar properties, we used the assortativity statistic. Specifically, we quantified degree assortativity by correlating nodal degree profiles on the ends of each edges (Barrat, Barthélemy, Pastor-Satorras, & Vespignani, 2004; Leung & Chau, 2007; Newman, 2002).

Finally, to capture the small-worldness organization of the network, we assessed clustering and characteristic path length. The clustering coefficient measures the tendency for a node’s neghbors to be connected with each other by counting the fraction of triangles around each node (Watts & Strogatz, 1998). In the present report we used a weighted and directed analogue of nodal clustering (Fagiolo, 2007; Onnela, Saramäki, Kertész, & Kaski, 2005). Characteristic path length describes the harmonic mean length of weighted shortest paths between all pairs of nodes in the network (Watts & Strogatz, 1998). To recover weighted shortest paths, we used a logarithmic weight to distance mapping, such that large edge weights correspond to short distances. Low characteristic path length suggests that the network is well configured to pass signals among constituent nodes. All measures were contrasted against a population of 1,000 randomly rewired networks with preserved in- and out-degree.

### Community Detection

Communities are groups of nodes with dense connectivity among each other. A Louvain-like greedy algorithm was used to identify a community assignment or partition that maximizes the quality function *Q* (Blondel, Guillaume, Lambiotte, & Lefebvre, 2008; Leicht & Newman, 2008):

$$Q=\frac{1}{m}\underset{i,j}{\sum }\left[A_{ij}-\gamma \frac{s_{i}^{out}s_{j}^{in}}{m}\right]\delta (c_{i},c_{j}),$$ (3)

where *A*_*ij*_ is the weight of connection between nodes *i* and *j*, $s_{i}^{out}$ and $s_{j}^{in}$ are the directed strengths of *i* and *j*, *m* is a normalizing constant, *c*_*i*_ is the community assignment of node *i*, and *δ*-function *δ*(*u*, *v*) is defined as 1 if *u* = *v* and 0 otherwise. The resolution parameter *γ* scales the importance of the null model and effectively controls the size of the detected communities: Larger communities are more likely to be detected when *γ* < 1 and smaller communities (with fewer nodes in each community) are more likely to be detected when *γ* > 1.

To detect stable community assignments, the algorithm was initiated 1,000 times at each value of the resolution parameter and consensus clustering was used to identify the most representative partition (Lancichinetti & Fortunato, 2012). This procedure was repeated for a range of resolutions *γ* = [0.5, 2.2]. We then quantified the similarity between pairs of consensus partitions (Rubinov et al., 2015). The procedure yielded three regimes/intervals > 0.2 with near-maximal mutual information (*γ* = 0.8, 1.4, 2.0), which we focus on in the present report.

Given a partition, we quantify the diversity of a node’s connections to multiple communities using the *participation coefficient* (Guimera & Amaral, 2005). The in-participation coefficient is defined as

$$p_{i}^{in}=1-\underset{c\in C}{\sum }{\left[\frac{s_{i}{\left(c\right)}^{in}}{s_{i}^{in}}\right]}^{2}$$ (4)

and the out-participation coefficient is defined as

$$p_{i}^{out}=1-\underset{c\in C}{\sum }{\left[\frac{s_{i}{\left(c\right)}^{out}}{s_{i}^{out}}\right]}^{2},$$ (5)

where *s*_*i*_ is the total strength of node *i*, *s*_*i*_(*c*) is the strength of *i* in community *c*, and the sum is over the set of all communities *C*. Nodes with a low participation coefficient are mainly connected with nodes in a single community, while nodes with a high participation coefficient have a diverse connection profile, with connections to multiple communities.

### Rich Club Detection

A rich club is defined as a set of high-degree nodes that are also densely interconnected with each other, above and beyond their degree (Colizza et al., 2006; Opsahl et al., 2008). The procedure is performed over a range of degrees *k*. At each level *k*, all nodes with degree ≤ *k* were removed from the network. The density of the remaining subgraph is termed the rich club coefficient *ϕ*(*k*). An identical procedure is performed in parallel on a population of 1,000 randomly rewired networks with preserved in- and out-degree sequences (Maslov & Sneppen, 2002), yielding a null distribution of randomized rich club coefficients *ϕ*_*rand*_(*k*). A *p* value at each *k* level can then be estimated as the proportion of *ϕ*_*rand*_(*k*) that are greater than the empirical *ϕ*(*k*). A consistent regime of statistically significant *ϕ*(*k*) at large *k* values suggests the presence of rich club organization.

### Motif Composition

Motifs are local connection patterns among a set of nodes and can be thought of as the building blocks of the network (Milo et al., 2002). For three nodes, there are 13 possible configurations, including cycles, open triangles, and so forth. Here we computed the frequency of each of the 13 three-node motifs, relative to their frequency in a population of 1,000 randomly rewired networks with preserved in- and out-degree sequence. A two-tailed *p* value, indexing how unexpectedly frequent or infrequent a particular motif is, was estimated by computing the proportion of null networks with frequencies greater or smaller than in the real network.

### Persistent Homology

Persistent homology is computed through a process of edge filtration, using the Python binding of Dionysus 2 (Giusti et al., 2016; Reimann et al., 2017; Sizemore et al., 2018, 2019). The filtration is applied to the negative-log-transformed (weight-to-distance transformed) network by adding one edge at a time, in order of decreasing original connection weight. Through the filtration process, we track the formation of cycles: structures that, when considered as a geometric object, form a closed shell with no boundary. To detect topological cavities, we note the appearance (birth, *ρ*_*birth*_) and disappearance (death, *ρ*_*death*_) of voids enclosed by cycles. Persistent cavities, having a long lifetime (*ρ*_*death*_ − *ρ*_*birth*_), are thought to be relatively important to the architecture of the network. The persistence of cavities in the empirical network is contrasted against a population of 100 randomly rewired networks with preserved in- and out-degree sequences.

## ACKNOWLEDGMENTS

The authors thank Vincent Bazinet, Golia Shafiei, Ross Markello, Justine Hansen, Laura Suarez, and Bertha Vazquez-Rodriguez for helpful comments and stimulating discussion.

## DATA AVAILABILITY

Marmoset connectome data were downloaded from the Marmoset Brain Architecture Project.

## SUPPORTING INFORMATION

Supporting information for this article is available at https://doi.org/10.1162/netn_a_00159. Code used to perform the reported analyses is available on GitHub (Liu, 2020; https://github.com/netneurolab/marmoset_connectome) under the BSD 3-Clause License.

## AUTHOR CONTRIBUTIONS

Zhen-Qi Liu: Conceptualization; Formal analysis; Visualization; Writing - Original Draft. Ying-Qiu Zheng: Formal analysis; Writing - Review & Editing. Bratislav Misic: Conceptualization; Supervision; Writing - Review & Editing.

## FUNDING INFORMATION

Bratislav Misic, Natural Sciences and Engineering Research Council of Canada, Award ID: RGPIN #017-04265). Bratislav Misic, Canada Research Chairs Program, Award ID: 950-232177. Bratislav Misic, Canada First Research Excellence Fund, Award ID: Healthy Brains for Healthy Lives.

## TECHNICAL TERMS

Clustering: The proportion of a node’s neighbors that are also neighbors (connected) with each other.
Hub: A node that is disproportionately well connected or central in the network.
Community: Subnetworks of brain areas with a greater than expected density of connections among each other.
Motif: Repeating subgraphs or patterns that form the building blocks of the whole network.
Strength: The total weight of connections that a node participates in.
Degree: The number of edges or connections that a node participates in.
Characteristic path length: The mean length of all shortest paths (minimum contiguous set of edges) between all pairs of nodes.
Participation coefficient: A measure of how evenly distributed a nodes connections are among the network’s communities (1 = maximum, 0 = minimum).
Rich club: A group of high-degree nodes that are disproportionately densely connected with each other.
Cavity: Encapsulated combinations of nodes where information is exchanged serially, rather than in parallel.
