# Supplementary material for: Network topology of the marmoset connectome
Source: Netw Neurosci. 2020 Dec 1;4(4):1181–96. doi: 10.1162/netn_a_00159 (PMC7781610; doi:10.1162/netn_a_00159)
Supplement: Supplementary file 1 [file netn-04-1181-s001.pdf]

Liu, Z., Zheng, Y. & Misic., B. (2020). Supporting information for "Network topology of the marmoset connectome." *Network Neuroscience*, *4*(4), 1181–1196. https://doi.org/10.1162/netn_a_00159

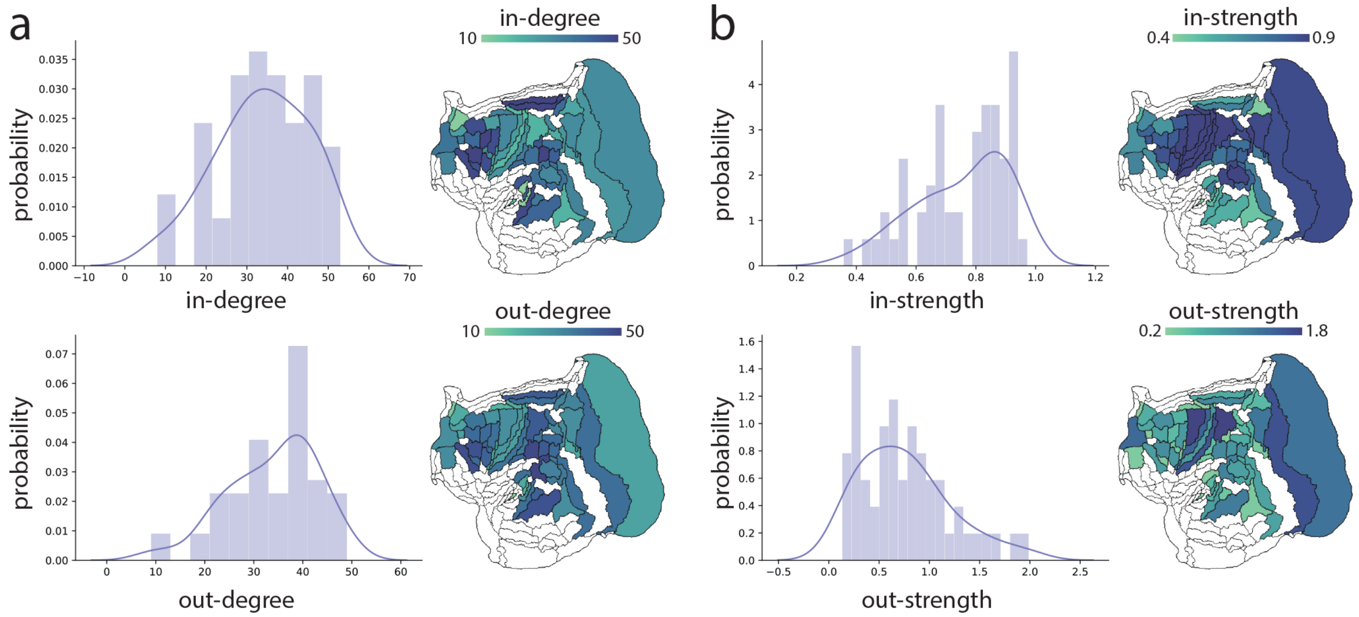

Figure S1. **Degree and strength distributions |** (a) Probability density of the binary in- and out-degree for each of the 55 nodes in the network. (b) Probability density of the weighted in- and out-strength for each of the 55 nodes in the network. All metrics are projected to a flatmap.
